# Abstracts and Condensations from German Journals

**Published:** 1871-12

**Authors:** Ch. Raushenberg

**Affiliations:** Atlanta, Georgia


### ABSTRACTS AND CONDENSATIONS FROM GERMAN JOURNALS.

BY CH. RAUSHENBERG, M.D., ATLANTA, GEORGIA.

### *War-Typhus and Dysentery. By Rud. Virchow.*

The distinguished author of this lengthy article on the causes and pathological anatomy of these camp diseases, calls the attention of the profession to the fact that the form of typhus occurring in the camps of contending armies—the camp-fever of English, the war-typhus of continental writers—is by no means always the typhus gravior, the petechial or spotted fever, which it has generally been considered to represent.

While the typhus epidemics of the Napoleonic wars, with but limited local exceptions, and those of the late Crimean war, represented typhus, (spotted fever, putrid fever), the disease as it occurred during the late Franco-Germanic war, most prominently within and around Metz and Paris during the siege of those cities, where larger numbers of contestants than ever before within historic recollection were exposed to severe privations for months, was abdominal typhus—the typhoid fever of American nosologists.

*The anatomical condition of the intestinal and mesenteric glands* is considered by him the only decisive criterion for the distinction between the two diseases. He denies that there is any form of typhus or typhoid fever in which hemorrhagic petechiæ constitute a pathognomic symptom, (although they frequently occur in both diseases in the latter stages), and admits no constant difference between the roseola of typhoid fever and the petechiæ of typhus, as it is usually considered to exist by many authors. In both cases, he says, it is principally a roseolous exanthem, caused by multiple capillary hyperæmia of the skin not attached to the hair follicles. In the commencement, the redness yields to pressure; later—probably in consequence of a partial effusion of the hæmatin and its imbibition into the tissues—a darker spot, which will only partially disappear under the pressure of the finger, is occasioned. In the typhus (the spotted fever), the exanthem is dark, thick and extensive, and roseola has, in later European epidemics, been occasionally observed on the face, the forehead, the palms of the hand, and feet. In typhoid fever, it is paler, weak, confined to certain regions, particularly the lower thoracic and upper abdominal ones; but occasionally, indubitable cases of typhoid fever occur in which the exanthem is strongly developed, and in such cases the spots are larger, of a darker color, and sometimes slightly elevated in the center, and then widely diffused over the surface. If it is taken into consideration, again, that it often happens in typhus that the exanthem is weak and scattered, it cannot be denied that typhoid fever, as well as typhus, must be looked upon as exanthematous diseases; but that the exanthem alone, without the consideration of other symptoms, offers no decisive, and in a strict sense of the word, no pathognomic symptom. A very strong eruption, particularly on the extremities, initiated and accompanied by a diffuse rash-like hyperæmia of the skin, must always create a strong suspicion of spotted fever, true typhus.

The anatomical condition of the intestinal and mesenteric glands can, unfortunately, only be ascertained with absolute certainty after death. The proof of the character of the epidemic prevailing during the Franco-Germanic war was furnished by a large number of post-mortem examinations. Extensive marrow-like, morbid vegetations on the lymphatic apparatuses of the lower small intestines, and partly of the large intestines, ulcers and cicatrices have generally been discovered. During life, we have no unmistakable evidences of these changes. The diarrhœa and meteorismus of the intestines are remarkable, particularly if fluid and gas are discovered principally at the affected part, the lower ileum and the cæcum, and produce the well-known ileo-cæcal noise; but even this noise is not specific for typhoid fever, as it is heard occasionally, in a very similar manner, in typhus.

The idea that the diarrhœa of typhoid fever is principally occasioned by the formation of ulcers in the intestines, as if the diarrhœal fluid were secreted from the surface of the ulcers, is also very erroneous. The number and size of the ulcerations has, as has been demonstrated previously, (Virchow's Archiv, vol. i., page 249), no direct connection with the diarrhœa, and the liquid discharges are by no means secretions of the ulcerated surfaces, but are caused by the concomitant intestinal catarrh, which generally extends far beyond the region of the ulcers. The intestinal affection of typhoid fever embraces two distinct and separate items: *the catarrh of the mucous membrane*, which extends along the whole surface of the small intestines into the stomach; and *the progressive vegetations of the lymphatic apparatus*, which occurs in the solitary and Peyer's follicles, (and also the mesenteric glands), principally in the lower portion of the ileum. Both kinds of changes are locally, as well as physiologically, different from each other.

Virchow contends that he discovered already, in 1848, that the *ulcer* is not the principal peculiarity of typhoid fever (abdominal typus), as ulceration is frequently prevented by a resorption of the deposit while it occupies the stage of marrow-like infiltration. (Virchow's Archiv, vol. ii., page 244.) C. E. E. Hoffman (*Untersuchungen uber die path anat., Veranderungen der Organe beim Abdominal Typhus, Leipzig*, 1869, page 62–109) endeavors to give a minute description of this process of resorption.

The opinion that the formation of ulcers is the regular and legitimate issue of the local typhoid process in the intestines is too common amongst practitioners; and medical writers, even if they speak of the possibility of this resorption, seem generally to entertain the idea, that this is merely an exception to the usual rule, deserving but little attention; hence, the period of ulceration is almost everywhere treated of as a regular and constant occurrence in the history of the disease.

Virchow declares this *resorption or resolution* as the most normal issue of the local process of typhoid fever, as much so as in pneumonia or peritonitis. The marrow-like infiltration of the lymphatic apparatus, however, is not a simple effusion, but a progressive accumulation of lymphatic elements (alike the lymphatic gland-cells), which commences first in the follicles, and extends through their surroundings even into the subserous tissue, and hence takes place in a heteroplastic manner. These changes in the follicles run strictly parallel with a like infiltration of the mesenteric glands. A fact of great interest is, that a resolution forms, without any doubt, the general rule in the mesenteric glands. Even in those cases where ulceration takes place in the intestines, these glands only exceptionally undergo a corresponding change; in the large majority of cases, they remain in this condition of marrowy infiltration, and then spontaneously return to a relatively normal state.

Where ulceration does take place—and observation and comparison of the diseased mesenteric glands in different cases will illustrate this more obviously than the diseased follicles alone—a metamorphosis of the enlarged and infiltrated parts, of a tuberculous, cheesy character, connected with inspissation and death of the elements, can be discovered and demonstrated. The product of this metamorphosis is the *scurf of the typhoid ulcer* (sphacelus typhosus), not the *ulcer itself*, because the latter is called into existence only after this scurf separates from the intestinal walls. It is, therefore, very plain that we ought to consider this issue of the local typhoid process one of *mortification*, *but not of simple ulceration.* If the typhoid deposit were made within the skin, the metamorphosis of it would have been classed under the head of gangrene long ago.

The changes of the scurf in the intestinal canal, under the decomposing influences of its contents, are much greater than those of the scurf when it occurs in the mesenteric glands. But a minute description of either must be omitted for want of space.

The further pathological changes round these gangrenous hearths consist in the formation of a line of demarcation and the accumulation of pus in consequence of inflammation of the tissues around the scurf. A separation of it from the surrounding tissues takes place; it falls off, and an intestinal ulcer or a furuncular abscess establishes itself, which can, if favorably located, cause partial peritonitis mesenterica, with exudative products. These can become adhesive; and thus this process leads either to final incapsulation of the scurf or to perforation. Solitary follicles, when thus diseased, form small groups of Peyer's glands, larger ulcers.

The typhoid deposit, in many places, undergoes the cheesy metamorphosis only in central spots—hence, forming ulcers with swollen ring-like edges. These ulcers generally cicatrize, and the inflated edges are generally removed by resorption. The most general and most frequent occurrence is, however, *that the larger portion of the different infiltrated spots do not undergo this metamorphosis at all, but are resolved in toto*, while only in a small number of places this change takes place, and gives rise to the formation of ulcers. The higher up in the ileum these infiltrated spots are situated, the less is their inclination to undergo the change which leads to ulceration.

In consideration of these clinical observations, Virchow is of the opinion, that in favorable cases of typhoid fever, where the intensity and duration of the disease are small, and where a prompt restoration to health takes place, that ulceration and cicatrization are not the prominent processes of the diseases; and he thinks it justifiable to admit a strong probability, that cases of typhoid fever occur in which all infliltrated spots are totally removed by resorption, without any further metamorphosis, and without the formation of any ulcers.

In relation to certain transitions of typhoid fever into dysentery, and *vice versa*, frequently reported from the seat-of-war, Virchow says that such statements must depend upon misconceptions, caused by a deficient knowledge of the nature of dysentery.

He distinguishes, anatomically speaking, two forms of this disease—*the catarrhal and the diphtheritic form.* The former always exists first, and can, under certain influences, be transferred into the latter.

In the first form, a simple catarrhal change of considerable extent exists like it does in typhoid fever, with the difference, that in the former disease the large intestines, in the latter the small intestines, are prominently affected. This catarrh produces, besides fluid secretions, those thick tenacious masses which are called mucous when they are transparent, fibrinous and croupous when they are turbid, but which in reality are of an albuminous character. With these products of the so-called white flux, bloody admixtures in various quantities are associated in the so-called bloody flux or red dysentery. Both secretions emanate from the unchanged mucous membrane, without any erosion or ulceration existing there. In this simple catarrhal dysentery, the glandular mucous membrane of the colon (with Lieberkühn's glands) is certainly very influential in defining the character of these secretions, as much so as the vascular villous surface of the small intestines is in defining those of typhoid fever and catarrh of the small intestines.

In the hemorrhagic catarrh—which occurs in a similar but less degree also in cholera—the tenesmus has undoubtedly a very defining influence by forcing, under severe spasmodic contractions of the intestines, the blood to the very extremities of the hyperæmic mucous surface, until the minute blood-vessels burst, allowing a small part of the blood to exude into the tissues, and a still larger portion to accumulate in the intestinal tube.

While in the hyperæmic catarrh, the red dysentery indicates a higher degree of excitement, irritability and hyperæmia, it is prognostically important to know that both catarrhal forms of this disease never create erosions or ulcerations as long as they remain acute, and, therefore, admit of prompt cure. The restitution in integrum is accomplished direct, comparatively speaking, *per primam intentionem.*

Whenever these forms become subacute or chronic, they also become easily complicated with follicular ulcerations of the large intestines, which form real abscesses, cavities containing pus. These abscesses, when they first break open, show an ulcer with a narrow fistulous orifice; while typhoid ulcerations commence not as a cavity, but as a line of demarcation around the enlarged, solid, sphacelous follicle, representing on the mucous membrane at first not an orifice, but a ring. The pus in typhoid ulcerations is entirely subordinate; in the follicular ulceration of chronic catarrhal dysentery, the only product.

The glass-like mucus, which appears in this disease amongst the alvine discharges in the form of small lumps, similar to the spawn of frogs or boiled grains of sago, is not a secretion of the follicles, but a product of Lieberkühn's glands, which, by gradual confluence, form in the later stages of this disease—mucous cysts (colitis cystica.) They are a much later product of the disease than the follicular abscesses. These glassy grains sometimes turn blue upon the addition of iodine, and seem to indicate, in some instances, an imperfect digestion of amylum substances.

The follicular ulceration mentioned above is not peculiarly or specifically belonging to chronic catarrhal dysentery, but occurs exactly in the same manner in other forms of intestinal catarrhs; for instance that of phthisis and scrofulosis.

There is no form of intestinal catarrh which produces a peculiar form of ulceration. Whenever ulcerations appear on a catarrhal surface, they are either the result of follicular abscesses or shallow diphtheric erosions.

In relation to the diphtheritic form of the disease, Virchow agrees with the views laid down by Rokitansky. (Lehrbuch, vol. iii., page 207.) Every dysentery commences as a catarrh in the first stage of the disease, and only becomes diphtheritic occasionally; but the catarrhal symptoms continue to the end, principally as diarrhœa. The diphtheritic patches, as such, do not secrete anything, but are rather dry, and only after the separation of the scurfs a moderate secretion of pus commences, which can continue or even increase after the whole process otherwise has ceased. The diphtheritic patches appear in a double form, either on the surface of a simple catarrhal, or on that of a hemorrhagically infiltrated mucous membrane.

In the first case, the diphtheritic scurf is gray, or whitish-gray, unless it has been changed by the coloring matter of the bile; in the second, it is of a reddish-brown, blackish or blackish-green color. As the diphtheritic process is one of death or mortification, the necessary and invariable issue is ulceration. Hence, *the catarrhal dysentery always occasions follicular, the diphtheria mucous ulcerations.* Sometimes follicular ulcerations occur in the latter form of dysentery, but they are never the result of follicular abscesses, but of firm diphtheritic infiltrations of the follicles, and these have a deceiving similarity to typhoid ulcers of the solitary follicles.

Diphtheritis frequently develops itself again on the same ulcerated spots, and therefore leads to continually increasing losses of substance, which, as the diphtheritic infiltration often affects the circumference of the originally ulcerated patches, gives to its ulcers a strictly phagedenic character.

After these explanations, it appears plain that no anatomical reason exists *why typhoid fever could not coexist with dysentery.* Both processes do exist in the same individual together. In the same manner in which diphtheritis angina oocurs with typhoid fever, so does dysentery; and even all three processes are found in the same individual at the same time. The dysentery in these cases belongs, generally, to the diphtheritic form. In all cases of that kind which have come to the observation of Virchow, the typhoid processes constituted the primary, the diphtheritic and dysenteric processes the secondary phenomenon; and he knows of no case where a dysentery had become complicated with typhoid fever, or much less of such where any reasons existed to opine that the dysenteric process had given origin to the typhoid one.

Virchow considers it a noteworthy fact for the comparative anatomy and future consideration of etiological questions relating to these camp diseases, that the pathological condition of the armies in the Franco-Germanic war approached very near to that of the armies in the late American war. In this war, typhus fever was hardly observed at all, and Woodard (Outlines of the Chief Camp Diseases of the United States Armies) is much inclined to look upon the few cases mentioned under this designation as the result of diagnostic errors. While typhus fever was thus very rare, typhoid fever and dysentery constituted one-fourth of all the diseases which occurred—a condition very much like that of the French and German armies in the late war, to which these statements refer. After a lengthy consideration of the alimentary, thermal and telluric influences to which the armies, particularly around Metz, were exposed, our author arrives at the conclusion that all these influences could and did only create diarrhœa, and that others—*either contagion or a local cause affecting a definite portion of the intestinal canal*—are required to produce dysentery. As he already endeavored to demonstrate previously, (Archiv, vol. v., page 352), he still holds that indurated and stagnating fæcal masses furnish this local cause of irritation producing dysentery. He says that diarrhœa, after and with constipation, frequently terminates in dysentery, as those places where the fæces accumulate are very prone to become diphtheritic; and that he has frequently seen, in dilated portions of the colon above intestinal strictures, where this stagnation takes place, not only diphtheritis, but numerous and deep follicular ulcerations. Under such circumstances, chemical decompositions with development of gases occur, and principally ammoniacal compositions are formed, and ammonia must certainly exercise a very decided irritating influence. In proof of the admissibility of this opinion are mentioned the extensive diphtheritis often connected with morbus Brightii, where urea finds its way largely into the intestinal secretions; the great inclination of catarrhal affections of the vesica, the urethra and the calices of the kidneys to produce ammoniacal decomposition of the urea and extensive diphtheritis, and the frequency of diphtheritis vagina in vesico-vaginal fistules.

It could be said that soldiers are more subject to diarrhœa than constipation; but it must not be forgotten that constipation and diarrhœa do not form such an irreconcilable contrast, as the retention of fæcal masses and diarrhœa do often coexist, and that fæcal retentions do often enough occur in soldiers when on the march or on advanced guard. Particularly the most of the wounded become subject to obstinate constipation as soon as they are confined to bed; and this, perhaps, explains the peculiar circumstance—which, however, might be referred to contagion—that dysentery made its appearance with a larger number of the severely wounded. This contagion does by no means exclude the appreciation of a number of auxiliary influences. Malaria, bad water, previous diseases, a cachectic condition, (scorbutic taint of our writers), can exercise their influence, and locality, season, age and sex must be taken into consideration. Hardly any one of these influences would be be self-sufficient for the production of dysentery; and even constipation has only a visible effect in its production, where alimentary or thermal influences have previously produced an intestinal catarrh.

With the abdominal typhus (typhoid fever), we find ourselves in a similar predicament—that is, we know indeed very little of its causes. Contagion is said, according to common experience, to be of small importance in the consideration of its origin, but still it cannot be excluded. (*Recherches sur les Analogies et les Différences qui Existent Entre le Typhus et la Fièvre Typhoïde, de Gaultier de Claubry.* Paris, 1838. Page 114.) The contamination of the soil by animal substances, particularly fæces, and the thus originating infection of the body by a peculiar poison, has, until yet, generally been considered the principal cause—which poison might either be attached to the drinking water or floating in the atmosphere. Overcrowded domicils, changes of locality, deficient nutrition, season, etc., cannot be left unconsidered; but we are accustomed to subordinate them to those more important influences mentioned above. All influences that will produce intestinal catarrh must certainly be taken into consideration as incidental causes. That all these influences congregate most favorably in a besieging army, needs no explanation. The removal of all filth, the cleaning of tents and houses, yards and surroundings, the surveillance of springs, wells and branches, should receive careful and prominent attention. A sanitary corps, under the direction of physicians, should always be engaged in the execution of strict camp-police regulations.

Virchow has closely observed several cases of typhoid fever which occurred under his immediate observation while in command of a sanitary train in the last war, but says that he is unable to furnish a clear etiology of these cases; also, he is in possession of correct and reliable data in relation to the conduct and residence of these patients from the time they left Berlin.

Finally, Virchow calls the attention of the profession to the fact, that all countries inhabited by Sclavonish nations, (Russians, Poles, and Bohemianites,) in the same manner in which they furnish a continued source of the cattle plague, furnish, also, a source for the repeated diffusion of typhus (spotted) fever.

Since the time of the morbus Hungaricus, the true typhus has followed nearly all armies which came from the East to Central Europe. The severest epidemic by which Germany and France has been visited in modern times was brought along by the French army on its retreat from Russia, in 1812, and the next epidemic of that kind originated when, on the soil of the Crimea, another French army came again in contact with a Sclavonian one. The epidemics in Upper Silesia in 1848, and in Eastern Prussia, in 1868, permitted manifold references to Sclavonic nationalities. The same is true of several smaller epidemics which have, since 1828, occurred in the province of Posen and the eastern frontiers of Prussia. The neighborhood of Poland seems to have as dangerous a signification for these countries as Ireland has for Great Britain, in relation to the origin of this disease.— *Virchow's Archiv*, *January*, 1871.

---

Carbolic Acid as a Local Anæsthetic.—Carbolic acid possesses wonderful powers as a local anæsthetic. If a portion of skin is covered with a cloth soaked in a saturated solution of carbolic acid for half an hour, and then a streak traced across the surface with a camel's-hair brush dipped in acid liquefied by one-twentieth its bulk of water, the skin may be divided along the course of the streak with a sharp scalpel, quite down to the subcutaneous cellular tissue, without causing any pain. Abscesses, whitloes and buboes may be opened with great advantage in this manner. Sometimes it is necessary, when making an incision through the integument, to brush out the wound made, with some liquefied acid, before making the incision deeper.—*Dr. J. H. Bill, in Braithwaite's Retrospect.*

